# Clinical Review of Antidiabetic Drugs: Implications for Type 2 Diabetes Mellitus Management

**DOI:** 10.3389/fendo.2017.00006

**Published:** 2017-01-24

**Authors:** Arun Chaudhury, Chitharanjan Duvoor, Vijaya Sena Reddy Dendi, Shashank Kraleti, Aditya Chada, Rahul Ravilla, Asween Marco, Nawal Singh Shekhawat, Maria Theresa Montales, Kevin Kuriakose, Appalanaidu Sasapu, Alexandria Beebe, Naveen Patil, Chaitanya K. Musham, Govinda Prasad Lohani, Wasique Mirza

**Affiliations:** ^1^GIM Foundation, Little Rock, AR, USA; ^2^University of Arkansas for Medical Sciences (UAMS), Little Rock, AR, USA; ^3^Christus Trinity Mother Frances Hospital, Tyler, TX, USA; ^4^University of Arkansas for Little Rock (UALR), Little Rock, AR, USA; ^5^Tutwiler Clinic, Tutwiler, MS, USA; ^6^Vanderbilt University, Nashville, TN, USA; ^7^Arkansas Department of Health, Little Rock, AR, USA; ^8^St. Vincent Infirmary, Little Rock, AR, USA; ^9^Baptist Hospital SpringHill, North Little Rock, AR, USA; ^10^The Wright Center for Graduate Medical Education, Scranton, PA, USA

**Keywords:** diabetes, clinical management, chronic, insulin, primary care

## Abstract

Type 2 diabetes mellitus (T2DM) is a global pandemic, as evident from the global cartographic picture of diabetes by the International Diabetes Federation (http://www.diabetesatlas.org/). Diabetes mellitus is a chronic, progressive, incompletely understood metabolic condition chiefly characterized by hyperglycemia. Impaired insulin secretion, resistance to tissue actions of insulin, or a combination of both are thought to be the commonest reasons contributing to the pathophysiology of T2DM, a spectrum of disease originally arising from tissue insulin resistance and gradually progressing to a state characterized by complete loss of secretory activity of the beta cells of the pancreas. T2DM is a major contributor to the very large rise in the rate of non-communicable diseases affecting developed as well as developing nations. In this mini review, we endeavor to outline the current management principles, including the spectrum of medications that are currently used for pharmacologic management, for lowering the elevated blood glucose in T2DM.

## Introduction

Diabetes mellitus (DM) is a complex chronic illness associated with a state of high blood glucose level, or hyperglycemia, occurring from deficiencies in insulin secretion, action, or both. The chronic metabolic imbalance associated with this disease puts patients at high risk for long-term macro- and microvascular complications, which if not provided with high quality care, lead to frequent hospitalization and complications, including elevated risk for cardiovascular diseases (CVDs) (1). The clinical diagnosis of diabetes is reliant on either one of the four plasma glucose (PG) criteria: elevated (i) fasting plasma glucose (FPG) (>126 mg/dL), (ii) 2 h PG during a 75-g oral glucose tolerance test (OGTT) (>200 mg/dL), (iii) random PG (>200 mg/dL) with classic signs and symptoms of hyperglycemia, or (iv) hemoglobin A1C level >6.5%. Recent American Diabetes Association (ADA) guidelines have advocated that no one test may be preferred over another for diagnosis. The recommendation is to test all adults beginning at age 45 years, regardless of body weight, and to test asymptomatic adults of any age who are overweight or obese, present with a diagnostic symptom, and have at least an additional risk factor for development of diabetes.

Furthermore, a condition called prediabetes or impaired fasting glucose (IFG), in which the fasting blood glucose is raised more than normal but does not reach the threshold to be considered diabetes (110–126 mg/dL), predisposes patients to diabetes, insulin resistance, and higher risk of cardiovascular (CV) and neurological pathologies (2, 3). Type 2 diabetes mellitus (T2DM) can co-occur with other medical conditions, such as gestational diabetes occurring during the second or third trimester of pregnancy or pancreatic disease associated with cystic fibrosis. T2DM may also be iatrogenically induced, e.g., by use of glucocorticoids in the inpatient setting or use of highly active antiretroviral agents like protease inhibitors and nucleoside reverse transcription inhibitors in HIV-positive individuals (4). Chemical diabetes or impaired glucose tolerance (IGT) may also develop with the use of thiazide diuretics, atypical antipsychotic agents, and statins (5, 6).

Type 2 diabetes mellitus is a common and increasingly prevalent disease and is thus a major public health concern worldwide. The International Diabetes Federation estimates that there are approximately 387 million people diagnosed with diabetes across the globe (7). According to Centers for Disease Control and Prevention, in 2012, 29.1 million adults, or 9.3% of the population, were identified with diabetes in the United States (US). Also in the same year, 86 million people had prediabetes condition and 15–30% of them developed into full-blown diabetes (8). In general, 1.4 million newly diagnosed cases in the US are being reported every year. If this trend continues, it is projected that in 2050 one in three Americans will have diabetes. Patients with diabetes have increased risk of serious health complications including myocardial infarction, stroke, kidney failure, vision loss, and premature death. Diabetes, with its associated side effects, remains the seventh leading cause of mortality in the US. The World Health Organization estimates that by 2030, mortality related to diabetes will double in number if not given deliberate attention (9). In addition, epidemiological studies report that diabetes causes more deaths in Americans every year compared to breast cancer and acquired immunodeficiency syndrome (AIDS) combined (10). The increasing trend in the incidence and prevalence of diabetes is worrisome and poses a great burden on medical costs and in our current healthcare system.

The ADA has released a range of recommendations called *Standards of Medical Care in Diabetes* to improve diabetes outcomes. The recommendations include cost-effective screening, diagnostic and therapeutic strategies to prevent, delay, or effectively manage T2DM and its life-threatening complications (11). Per recommendations of ADA and other organizations, modern approaches to diabetes care should involve a multidisciplinary team of health professionals working in tandem with the patient and the family (2). The primary aim of these approaches is to obtain optimal glycemic control through dietary and lifestyle modifications and appropriate medications along with regular blood glucose level monitoring. The burden of diabetes can be potentially reduced if the standard of care is implemented as well as patients’ compliance and participation is clinically implemented.

The traditional presentations of T2DM occurring only in adults and type 1 diabetes mellitus (T1DM) only in children are not entirely correctly representative, as both diseases occur in both age groups. Occasionally, patients with T2DM may develop the morbid complication of diabetic ketoacidosis (DKA) (12). Children with T1DM typically present with polyuria and polydipsia and approximately one-third of them present with DKA, which may also be the first presenting feature (12). The onset of T1DM may be variable in adults, and they may not present with the classic symptoms that are seen in children. The true diagnosis may become apparent with disease progression. The heterogeneity of the presentations should be kept in mind while caring for the patient with T2DM.

The scope of this review encompasses current clinical guidelines on the pharmacological management of T2DM.

## Clinical Diagnosis of Type 2 Diabetes

Diabetes may be identified in low-risk individuals who have spontaneous glucose testing during routine primary clinical care, in individuals examined for diabetes risk assessment, and in frankly symptomatic patients. Early diagnosis of T2DM can be accomplished through blood tests that measure PG levels. FPG is the most common test to detect diabetes: a level of ≥126 mg/dL or 7.0 mmol/L confirmed by repeating the test on another clinic visit effectively diagnoses the disease. This test requires fasting for at least the previous 8 h and generates enhanced reliability when blood is drawn in the morning. Another criterion is the 2 h PG of ≥200 mg/dL or 11.1 mmol/L in a patient presenting with the traditional symptoms of diabetes such as polyuria, polydipsia, and/or unexplained weight loss. A positive 2-h OGTT will show a PG level of ≥200 mg/dL or 11.1 mmol/L after a glucose load containing 75 g of glucose solution in water. Two-hour PG OGTT is not commonly used in the clinic because, although it is more sensitive than FPG test, it is less convenient and more expensive for patients. Additionally, this test holds less relevance in routine follow-ups after confirmed diagnosis of diabetes is obtained.

In the past, the glycated hemoglobin (HbA1C) test was used mainly to monitor the adequacy of glycemic management and has strong predictive value for diabetes complications (13). HbA1C is a chronic marker of hyperglycemia and reflects patient’s blood glucose level over a period of 3–4 months, coinciding with the lifespan of the red blood cells (RBCs). However, in 2009 after its standardization, the International Expert Committee recommended it to be used in diagnosing T2DM but not in T1DM and gestational diabetes (2). HbA1C level is reported in percentages, and a normal level is below 5.7%. The main advantage of the HbA1C test over other blood glucose tests is the convenience it offers to patients; it does not require fasting and can be done at any time of the day. However, this test is more expensive and may not be readily available in certain locations, which may limit its usefulness (14, 15). HbA1C may be inaccurate in conditions such as anemia, hemolysis, and other hemoglobinopathies like sickle cell disease and hemoglobin (Hb) variants like HbC, HbE, and HbD, as well as elevated fetal hemoglobin. Thus, HbA1C assay in people of South Asian, Mediterranean, or African origin merit taking these issues into account (16). In conditions associated with increased RBC breakdown, such as in the advanced trimesters of pregnancy, recent hemorrhage, intravascular hemolysis or transfusion or erythropoietin treatment, only blood glucose estimation should be used to diagnose diabetes. There are limited data supporting the use of A1C in diagnosing T2DM in children and adolescents. Although A1C is not routinely suggested for diagnosis of diabetes in children with cystic fibrosis or symptoms that portend development of acute onset of T1DM, the ADA recommends HbA1C for diagnosis of T2DM in children and adolescents.

In order to accurately diagnose diabetes and in the absence of frank hyperglycemia (PG > 200 mg/dL) or hyperglycemic crisis, it is useful to repeat the same diagnostic test for confirmation. In situations where there are two different tests with conflicting results, the test which is positive should be repeated and a diagnosis of diabetes is made after a confirmatory test has been done (2). For individuals whose test result/s returned negative for diabetes, repeat testing at 3-year intervals is suggested (17).

The ADA and American Association of Clinical Endocrinologists recommend screening for prediabetes beginning at age 45 years or earlier for asymptomatic individuals with strong risk factors such as obesity (BMI ≥ 25 kg/m^2^), hypertension and family history (first degree relative with diabetes) (18). IFG level of 100–125 mg/dL (5.6–6.9 mmol/L), IGT with a 2-h OGTT PG level between 140 and 199 mg/dL (7.9–11.0 mmol/L), or an HbA1C of 5.7–6.4% indicates prediabetes. Patients with an HbA1C level of >6% are considered high risk of developing diabetes, and early detection is necessary to prevent adverse outcomes. Patients diagnosed with prediabetes can be retested after a year; however, without proper intervention 70% of individuals diagnosed with prediabetes are most likely to progress to diabetes in 10 years or even less, depending on their risk factors (18). It is also important to note that prediabetes may be associated with obesity, dyslipidemia, and hypertension; therefore, lifestyle changes such as healthy diet, physical activity, and cessation of smoking, in addition to the introduction of pharmacological agents, are deemed important to stop or delay the timeline of development of diabetes.

## Clinical Management of Type 2 Diabetes

Comprehensive care for a patient with diabetes requires an initial evaluation of the patient’s risk factors, the presence or absence of diabetes complications, and initial review of previous treatment/s (2). This will enable the healthcare providers to optimally manage patients with either prediabetes or diabetes. The cornerstones of diabetes management include lifestyle intervention along with pharmacological therapy and routine blood glucose monitoring.

### Lifestyle Measures

Clinical trials have shown that lifestyle modifications are cost-effective in preventing or delaying the onset of diabetes, with approximately 58% reduction in risk in 3 years (19). It is highly recommended by the ADA that patients with IGT, IFG or HbA1C level of 5.7–6.4% be counseled on lifestyle changes such as diet and exercise. On the other hand, for patients who are already diagnosed with diabetes, nutrition advice provided by a registered dietitian is recommended. A goal of moderate weight loss (≈7% of body weight) is an important component in the prevention and treatment of diabetes, as it can improve blood glucose levels, and can also positively impact blood pressure and cholesterol levels (19). Weight loss can be achieved through a healthy balanced diet, with control of total calories and free carbohydrates. However, for patients with diabetes adhering to a low carbohydrate diet, they should be informed on possible side effects such as hypoglycemia, headache and constipation (20). Other studies have suggested consumption of complex dietary fiber and whole grains to improve glycemic control (2, 21).

Studies show that exercise can improve glycemic control (lower HbA1C level by 0.66%), with or without significant decrease in body weight, and improve the total well-being of patients (22). It is considered an integral part in the prevention and management of both prediabetes and diabetes. According to the U.S. Department of Health and Human Services, adults ≥18 years of age should do a minimum of 150 min/week of moderate intensity exercise (e.g., walking at a 15- to 20-min mile pace) or 75 min/week of vigorous physical activity (e.g., running, aerobics) spread over at least 3 days/week with no more than two consecutive days without exercise to achieve maximum benefits (2, 18). For patients ≤18 years old, 60 min of physical activity every day is adequate.

Other lifestyle measures that need to be considered in the treatment plan for patients with diabetes are moderate alcohol consumption (≤1 drink for women, ≤2 drinks/men) and reduction in sodium intake especially in patients with comorbidities such as hypertension, habitual tobacco use, and lacking immunizations (influenza, diphtheria, pertussis, tetanus, pneumococcal, and hepatitis B). Consumption of alcohol, especially in a fasted state, can precipitate life-threatening hypoglycemia and coma and should be explicitly counseled to patients during their visits (23). Moreover, patient education, counseling, and psychosocial support are very important to successfully combat the deleterious effects of diabetes.

### Pharmacologic Management

An “ominous octet” that leads to hyperglycemia, which occur in isolation or in combination, has been proposed for eight pathophysiological mechanisms underlying T2DM (24). These include (i) reduced insulin secretion from pancreatic β-cells, (ii) elevated glucagon secretion from pancreatic α cells, (iii) increased production of glucose in liver, (iv) neurotransmitter dysfunction and insulin resistance in the brain, (v) enhanced lipolysis, (vi) increased renal glucose reabsorption, (vii) reduced incretin effect in the small intestine, and (viii) impaired or diminished glucose uptake in peripheral tissues such as skeletal muscle, liver, and adipose tissue. Currently available glucose-lowering therapies target one or more of these key pathways.

Good glycemic control remains the main foundation of managing T2DM. Such approaches play a vital role in preventing or delaying the onset and progression of diabetic complications. It is important that a patient-centered approach should be used to guide the choice of pharmacological agents. The factors to be considered include efficacy, cost, potential side effects, weight gain, comorbidities, hypoglycemia risk, and patient preferences. Pharmacological treatment of T2DM should be initiated when glycemic control is not achieved or if HbA1C rises to 6.5% after 2–3 months of lifestyle intervention. Not delaying treatment and motivating patients to initiate pharmacotherapy can considerably prevent the risk of the irreversible microvascular complications such as retinopathy and glomerular damage (25). Monotherapy with an oral medication should be started concomitantly with intensive lifestyle management.

**Figure 1:**
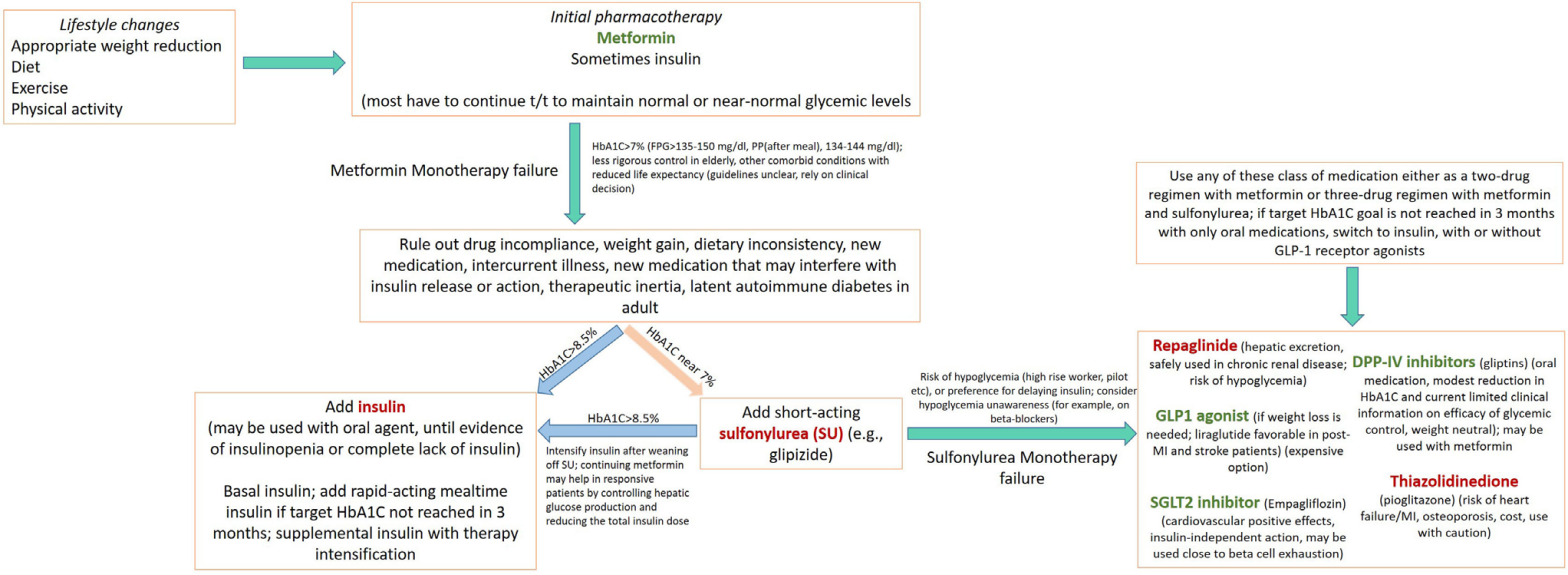
**Flow chart depicting an algorithm for use of drug regimen in treating diabetes mellitus Several concepts presented here are adapted from American Diabetes Association/European Association for the Study of Diabetes (27–30)**. Medications in green, causes weight loss; in red, causes weight gain.

The major classes of oral antidiabetic medications include biguanides, sulfonylureas, meglitinide, thiazolidinedione (TZD), dipeptidyl peptidase 4 (DPP-4) inhibitors, sodium-glucose cotransporter (SGLT2) inhibitors, and α-glucosidase inhibitors. If the HbA1C level rises to 7.5% while on medication or if the initial HbA1C is ≥9%, combination therapy with two oral agents, or with insulin, may be considered (2, 26). Though these medications may be used in all patients irrespective of their body weight, some medications like liraglutide may have distinct advantages in obese patients in comparison to lean diabetics (see below). A schematic of currently approved medications for T2DM is summarized in Table 1. A flowchart for guiding clinical decision making is presented in Figure 1.

**Table 1:** **Pharmacological agents for glycemic control**.

Class of antidiabetic medication (route of administration)	Representative agents	Mechanism of action	T1/2 and metabolism	HbA1C reduction (%)	Risk of hypoglycemia	Effect on body weight	Metabolic alterations	Cardiovascular (CV) benefit and risk	Other adverse effects/additional comments
Biguanide (o)	Metformin	Insulin sensitizer Numerous effects on inhibition of hepatic glucose production	5 h; unmetabolized, renal excretion	1–2	None	Mild weight loss due to anorectic effect	Lactic acidosis (very rare) May cause nausea/vomiting or diarrhea after introduction, which may result in electrolyte or pH alterations	Reduce MI by 39% and coronary deaths by 50% (UKPDS)	Vitamin B12 deficiency, which may cause anemia and neuropathy (risk in elderly) Very safe drug, but stop metformin if creatinine >1.5 mg/dL in males and >1.4 mg/dL in females

Dipeptidyl peptidase 4 (DPP-IV) inhibitor (o)	Sitagliptin Saxagliptin Vidagliptin Linagliptin Alogliptin	Inhibition of degradation of GLP	Excreted by kidneys (except linagliptin) (needs dose reduction in renal failure)	0.5–0.8	Low			Long-term trials to assess CV risk; decreases postprandial lipemia, however, may cause CHF by degradation of BNP	Pancreatitis Upper RTI infection

Sodium-glucose cotransporter (SGLT2) inhibitor (o)	Canagliflozin Dapagliflozin Empagliflozin	Glucosuria due to blocking (90%) of glucose reabsorption in renal PCT; insulin-independent mechanism of action			Low			Positive CV effect due to reduction of sodium and uric acid absorption and reduction of BP	Ketoacidosis (rare) Genital mycosis May increase LDLc Bone fractures

Insulin (p)	Short-acting Regular (R) (Humulin R, Novolin R) Intermediate NPH (N) Long-acting Insulin glargine (Lantus) Insulin detemir (Levemir) Insulin degludec (Tresiba) Rapid-acting Humalog (Lispro) Novolog (Aspart) Glulisine (Apidra) Pre-mixed 75% insulin lispro protamine/25% insulin lispro (Humalog Mix 75/25) 50% insulin lispro protamine/50% insulin lispro (Humalog Mix 50/50) 70% insulin lispro protamine/30% insulin aspart (Novolog 70/30) 70% NPH insulin/30% regular	Activation of insulin receptors and downstream signaling in multiple sensitive tissues	30 min-1 r (onset of action) Peak 2–5 h Duration of action 8 h 1.5–4 h (onset of action) Peak 4–12 h Duration of action 24 h 0.8–4 h (onset of action) Peak minimal Duration of action 24 h 10–30 min (onset of action) Peak 30 min–3 h Duration of action 3–5 h 5–15 min (onset of action) Peak dual Duration of action 10–16 h 30–60 min (onset of action) Peak dual Duration of action 10–16 h	1–2.5	Prominent	Weight gain		HF if used in combination with thiazolidinediones (TZD)	Lipoatrophy and lipohypertrophy at sites of injection Allergy to injection components Levemir Food and Drug Administration -approved for gestational diabetes mellitus

GLP-1 agonists (p)	Liraglutide Exenatide Dulaglutide	Activate GLP1 receptor Increased insulin secretion, decreased glucagon, delayed gastric emptying, increased satiety	24 h 4–6 h (short acting) 7 days (long acting, extended release) 7 days	0.5–1.5	No [risk if used in combination with sulfonylureas (SU)]	Weight loss		Reduce CV risk	Nausea, vomiting, pancreatitis, C cell tumor of thyroid (contraindicated in MEN type 2)

SU (o)	Glimepiride Glipizide Glyburide	Insulin secretion		1–2	Prominent (severe in renal failure)	Weight gain		Increased cardiovascular disease risk, mainly due to hypoglycemia	Use beta-blockers with caution

TZD (o)	Rosiglitazone Pioglitazone	True insulin sensitizer		0.5–1.4		Weight gain		Cardiac failure, pedal edema	Bladder cancer; fractures

*O, oral; p, parenteral; iv, intravenous; sc, subcutaneous*.

#### Biguanide

The discovery of biguanide and its derivatives for the management of diabetes started in the middle ages. *Galega officinalis*, a herbaceous plant, was found to contain guanidine, galegine, and biguanide, which decreased blood glucose levels (31). Metformin is a biguanide that is the main first-line oral drug of choice in the management of T2DM across all age groups. Metformin activates adenosine monophosphate-activated protein kinase in the liver, causing hepatic uptake of glucose and inhibiting gluconeogenesis through complex effects on the mitochondrial enzymes (31). Metformin is highly tolerated and has only mild side effects, low risk of hypoglycemia and low chances of weight gain. Metformin is shown to delay the progression of T2DM, reduce the risk of complications, and reduce mortality rates in patients by decreasing hepatic glucose synthesis (gluconeogenesis) and sensitizing peripheral tissues to insulin (31). Furthermore, it improves insulin sensitivity by activating insulin receptor expression and enhancing tyrosine kinase activity. Recent evidence also suggest that metformin lowers plasma lipid levels through a peroxisome proliferator-activated receptor (PPAR)-α pathway, which prevents CVDs (31). Reduction of food intake possibly occurs by glucagon-like peptide-1 (GLP-1)-mediated incretin-like actions. Metformin may thus induce modest weight loss in overweight and obese individuals at risk for diabetes.

Once ingested, metformin (with a half-life of approximately 5 h) is absorbed by organic cation transporters and remains unmetabolized in the body and is widely distributed into different tissues such as intestine, liver, and kidney. The primary route of elimination is via kidney. Metformin is contraindicated in patients with advanced stages of renal insufficiency, indicated by a glomerular filtration rate (GFR) <30 mL/min/1.73 m^2^ (32). If metformin is used when GFR is significantly diminished, the dose should be reduced and patients should be advised to discontinue the medication if nausea, vomiting, and dehydration arises from any other cause (to prevent ketoacidosis). It is important to assess renal function prior to starting this medication.

Metformin has an excellent safety profile, though may cause gastrointestinal disturbances including diarrhea, nausea, and dyspepsia in almost 30% of subjects after initiation. Introduction of metformin at low doses often improve tolerance. Extended release preparations seldom cause any gastrointestinal issues. Very rarely, metformin may cause lactic acidosis, mainly in subjects with severe renal insufficiency. Another potential problem arising from the use of metformin is the reduction in the drug’s efficiency as diabetes progresses. Metformin is highly efficient when there is enough insulin production; however, when diabetes reaches the state of failure of β-cells and resulting in a type 1 phenotype, metformin loses its efficacy.

Metformin can cause vitamin B12 and folic acid deficiency (33). This needs to be monitored, especially in elderly patients. Though very rare, in patients with metformin intolerance or contraindications, an initial drug from other oral classes may be used. Although trials have compared dual therapy with metformin alone, few directly compare drugs as add-on therapy. A comparative effectiveness meta-analysis suggests that overall each new class of non-insulin medications introduced in addition to the initial therapy lowers A1C around 0.9–1.1%. An ongoing Glycemia Reduction Approaches in Diabetes: A Comparative Effectiveness Study (GRADE) has compared the effect of four major drug classes (sulfonylurea, DPP-4 inhibitor, GLP-1 analog, and basal insulin) over 4 years on glycemic control and other psychosocial, medical, and health economic outcomes (34). Though it will be a welcome development for introduction of oral agents for metformin for gestational diabetes, current FDA regulations do not support it.

#### Incretin Mimetics

Incretin effect is the difference in insulin secretory response from an oral glucose load in comparison to glucose administered intravenously. The incretin effect is responsible for 50–70% of total insulin secretion after oral glucose intake (35). The two naturally occurring incretin hormones that play important roles in the maintenance of glycemic control: glucose-dependent insulinotropic polypeptide (GIP, or incretin) and glucagon-like peptide (GLP-1); these peptides have a short half-life, as these are rapidly hydrolyzed by DPP-4 inhibitors within 1½ min. In patients with T2DM, the incretin effect is reduced or absent. In particular, the insulinotropic action of GIP is lost in patients with T2DM. Incretins decrease gastric emptying and causes weight loss. Because of impact on weight loss, these medications may find increasing use in diabesity.

Targeting the incretin system has become an important therapeutic approach for treating T2DM. These two drug classes include GLP-1 receptor agonists and DPP-4 inhibitors. Clinical data have revealed that these therapies improve glycemic control while reducing body weight (specifically, GLP-1 receptor agonists) and systolic blood pressure in patients with T2DM (36). Furthermore, hypoglycemia is low (except when used in combination with a sulfonylurea) because of their glucose-dependent mechanism of action.

##### GLP-1 Receptor Agonists

The currently GLP-1 receptor agonists available are exenatide and liraglutide. These drugs exhibit increased resistance to enzymatic degradation by DPP4. In young patients with recent diagnosis of T2DM, central obesity, and abnormal metabolic profile, one should consider treatment with GLP-1 analogs that would have a beneficial effect on weight loss and improve the metabolic dysfunction. GLP-1 analogs are contraindicated in renal failure.

###### Exenatide

Exenatide, an exendin-4 mimetic with 53% sequence homology to native GLP-1, is currently approved for T2DM treatment as a single drug in the US and in combination with metformin ± sulfonylurea. Because of its half-life of 2.4 h, exenatide is advised for twice-daily dosing. Treatment with 10 µg exenatide, as an add-on to metformin, resulted in significant weight loss (−2.8 kg) in comparison to patients previously treated with metformin alone. Exenatide is generally well tolerated, with mild-to-moderate gastrointestinal effects being the most common adverse effect.

###### Liraglutide

Liraglutide is a GLP-1 analog that shares 97% sequence identity to native GLP-1. Liraglutide has a long duration of action (24 h). Liraglutide causes 1.5% decrease in A1C in individuals with type 2 diabetes, when used as monotherapy or in combination with one or more selected oral antidiabetic drugs. Liraglutide decreases body weight; the greatest weight loss resulted from treatment with liraglutide in combination with combined metformin/sulfonylurea (−3.24 kg with 1.8 mg liraglutide). Liraglutide also diminishes systolic pressure (mean decrease −2.1 to −6.7 mmHg) (37). Liraglutide is well tolerated, with only nausea and minor hypoglycemia (risk increased with use of sulfonylureas).

Serum antibody formation was very low in patients treated with once-weekly GLP-1 receptor agonists. The formation of these antibodies did not decrease efficacy of their actions on blood glucose lowering.

##### DPP-4 Inhibitors

Dipeptidyl peptidase 4 inhibitors include sitagliptin, saxagliptin, vidagliptin, linagliptin, and alogliptin. These medications may be used as single therapy, or in addition with metformin, sulfonylurea, or TZD. This treatment is similar to the other oral antidiabetic drugs. The gliptins have not been reported to cause higher incidence of hypoglycemic events compared with controls.

Dipeptidyl peptidase 4 inhibitors impact postprandial lipid levels. Treatment with vidagliptin for 4 weeks decreases postprandial plasma triglyceride and apolipoprotein B-48-containing triglyceride-rich lipoprotein particle metabolism after a fat-rich meal in T2DM patients who have previously not been exposed to these medications. In diabetic patients with coronary heart disease, it was demonstrated that treatment with sitagliptin improved cardiac function and coronary artery perfusion.

The three most commonly reported adverse reactions in clinical trials with gliptins were nasopharyngitis, upper respiratory tract infection, and headache. Acute pancreatitis was reported in a fraction of subjects taking sitagliptin or metformin and sitagliptin. An increased incidence of hypoglycemia was observed in the sulfonylurea treatment group.

In the elderly, DPP-4 inhibitors lower blood glucose but have minimal effect on caloric intake and therefore less catabolic effect on muscle and total body protein mass. In decreased doses, DPP-4 inhibitors are considered safe in patients with moderate to severe renal failure.

#### SGLT2 Inhibitors

Sodium-glucose cotransporter inhibitors are new classes of glucosuric agents: canagliflozin, dapagliflozin, and empagliflozin. SGLT2 inhibitors provide insulin-independent glucose lowering by blocking glucose reabsorption in the proximal renal tubule by inhibiting SGLT2 (38).

Because of glucose-independent mechanism of action, these drugs may be effective in advanced stages of T2DM when pancreatic β-cell reserves are permanently lost. These drugs provide modest weight loss and blood pressure reduction.

Urinary tract infections leading to urosepsis and pyelonephritis, as well as genital mycosis, may occur with SGLT2 inhibitors. SGLT2 inhibitors may rarely cause ketoacidosis. Patients should stop taking their SGLT2 inhibitor and seek medical attention immediately if they have symptoms of ketoacidosis (frank nausea or vomiting, or even non-specific features like tiredness or abdominal discomfort).

#### Insulin

If non-insulin monotherapy like metformin at the maximum tolerated dose does not achieve or maintain the A1C target over 3 months, then a second oral agent may be added to the regimen, a GLP-1 receptor agonist, or basal insulin. Insulin therapy (with or without additional agents) should be introduced in patients with newly identified T2DM and frankly symptomatic (catabolic features like weight loss, ketosis or features of hyperglycemia including polyuria/polydipsia) and/or severely elevated blood glucose levels [≥300–350 mg/dL (16.7–19.4 mmol/L)] or A1C [≥10–12%] (11).

The clinical picture of T2DM and its therapies should be regularly and objectively elaborated to patients. Many subjects with T2DM shall require insulin therapy sometime during the course of the disease. For patients with T2DM with inadequate target glycemic goals, insulin therapy should not be postponed. Providers should advocate insulin as a therapy in a complete non-judgmental, empathetic, and non-punitive approach to ensure superior quality of adherence. Self-monitoring of blood glucose (SMBG) (discussed below) contributes to significant improvement of glycemic control in patients with T2DM initiating insulin. Close and frequent monitoring of the patient is needed for any dose titration to achieve target glycemic goals, as well as to prevent hypoglycemia.

*Basal insulin* is the initial insulin regimen, beginning at 10 U or 0.1–0.2 U/kg, depending on the hyperglycemia severity (titrating by 2–3 U every 4–7 days till glycemic goal is reached). Use of basal insulin greater than 0.5 U/kg indicates the need for use of an additional agent. Basal insulin is usually added to oral metformin and possibly one additional non-insulin agent like DPP-4 or SGLT-2 inhibitor. *NPH (neutral protamine Hagedorn) insulin* carries low risk of hypoglycemia in individuals without any significant past history, and is low cost. Newer, longer acting, basal insulin analogs have superior pharmacodynamic profiles, delayed onset and longer duration of action but low risk of hypoglycemia, albeit at higher costs. Concentrated basal insulin preparations such as *U-500 regular* is five times more potent per volume of insulin (i.e., 0.01 mL ~5 U of U-100 regular) than *U-100 regular*. *U-300 glargine* and *U-200 degludec* are other potent, ultra-long acting preparations.

If *basal insulin* contributes to acceptable fasting blood glucose, but A1C persistently remains above target, mealtime insulin may be added. *Rapid-acting insulin analog (lispro, aspart, or glulisine)* may be used and administered just before meals. The glucose levels should be monitored before meals and after the injections. Another approach to control the periprandial glucose excursions may be to add twice-daily *premixed (or biphasic) insulin analogs (70/30 aspart mix, 75/25 or 50/50 lispro mix*). The total present insulin dose may be computed and then one-half of this amount may be administered as basal and the other half during mealtime, the latter split equally between three meals. Regular human insulin and human NPH–Regular premixed formulations (70/30) are less expensive alternatives to rapid-acting insulin analogs and premixed insulin analogs, respectively, but their unpredictable pharmacodynamic profiles make them inadequate to cover postprandial glucose changes.

Sometime, *bolus insulin* needs to be administered in addition to basal insulin. Rapid-acting analogs are used as bolus formulations due to their prompt onset of action. Insulin pump (continuous subcutaneous insulin infusion) may be used instead to avoid multiple injections. Often, patients and physicians are reluctant to intensify therapy due to the fear of hypoglycemia, regimen complexity, and increased multiple daily injections. There is a need for a flexible, alternative intensification option taking into account individual patient considerations to achieve or maintain individual glycemic targets. An ideal insulin regimen should mimic physiological insulin release while providing optimal glycemic control with low risk of hypoglycemia, weight gain, and fewer daily injections.

Inhaled insulin (Technosphere insulin-inhalation system, Afrezza) is now available for prandial use. However, the dosing range is limited. Use of inhaled insulin requires pulmonary function testing prior to and after starting therapy. It is contraindicated in subjects with asthma or other lung diseases.

During insulin therapy, sulfonylureas, DPP-4 inhibitors, and GLP-1 receptor agonists are stopped once more complex insulin regimens beyond basal insulin are used. In patients with inadequate blood glucose control, especially if requiring escalating insulin doses, TZDs (usually pioglitazone) or SGLT2 inhibitors may be added as adjunctive therapy to insulin.

Insulin injections can cause weight gain or loss. Insulin drives potassium into the cell and can cause hypokalemia. Components of the insulin preparation have the potential to cause allergy. Insulin injections, along with the use of other drugs like TZDs, can precipitate cardiac failure.

Stressful events like illness, surgery, and trauma can impede glycemic control and may lead to development of DKA or non-ketotic hyperosmolar state, life-threatening conditions, which merits immediate medical attention. Any condition that deteriorates glycemic control necessitates more frequent monitoring of blood glucose in an inpatient setting; ketosis-prone patients also require urine or blood ketone monitoring. If accompanied by ketosis, vomiting, or altered mental status, marked hyperglycemia requires hospital admission. The patient treated with non-insulin therapies or medical nutrition therapy alone may require insulin. Patient must be aggressively hydrated and infections should be controlled.

Without adequate treatment, prolonged hyperglycemia can cause glucose toxicity that can progressively impair insulin secretion. Initiation of insulin therapy is critical to reverse the toxic effect of high blood glucose levels on the pancreas. Once persistent glycemic control is achieved, insulin can be tapered off and replaced with oral medications. At some point in the management of T2DM, β-cell reserves are exhausted, with phenotypic reversal to a T1DM kind of pathophysiological situation. Meticulous follow-up may identify such states and then the need for continued reliance on insulin therapy may be carefully explained to the patients.

Weight gain can raise a barrier to the use of insulin in T2DM. In the United Kingdom Prospective Diabetes Study (UKPDS) study, patients gained 6 kg with insulin therapy, when compared with 1.7–2.6 kg weight gain with sulfonylureas (39). More recently, the combination of GLP-1 receptor agonists and insulin has been useful in tackling the weight gain associated with insulin and circumventing the need for high doses in the presence of significant insulin resistance. Lipoatrophy with insulin injections is not seen now; however, lipohypertrophy due to failure to change the subcutaneous injection sites is still a common cause of poor insulin absorption and suboptimal glycemic control.

In the Action to Control Cardiovascular Risk in Diabetes trial, aggressive treatment of T2DM patients with higher CV risk was associated with higher all-cause and CV mortality. *Post hoc* analyses could not find correlation with faster rates of reduction of glucose, hypoglycemia, or specific drugs as the causes underlying this finding. Exposure to injected insulin was hypothesized to increase CV mortality. However, after adjustment for baseline covariates, no significant association of insulin dose with CV death remained (40). Older patients with cognitive dysfunction may not benefit from intensive therapy. Furthermore, hypoglycemia in the elderly may cause cardiac ischemia, arrhythmia, myocardial infarction, and sudden death (41).

#### Sulfonylureas

Sulfonylureas lower blood glucose level by increasing insulin secretion in the pancreas by blocking the K_ATP_ channels. They also limit gluconeogenesis in the liver. Sulfonylureas decrease breakdown of lipids to fatty acids and reduce clearance of insulin in the liver (42). Sulfonylureas are currently prescribed as second-line or add-on treatment options for management of T2DM. They are divided into two groups: first-generation agents, which includes chlorpropamide, tolazamide, and tolbutamide, and second-generation agents, which includes glipizide, glimepiride, and glyburide. The first-generation sulfonylureas are known to have longer half-lives, higher risk of hypoglycemia, and slower onset of action, as compared to second-generation sulfonylureas. Currently, in clinical practice, second-generation sulfonylureas are prescribed and more preferred over first-generation agents because they are proven to be more potent (given to patients at lower doses with less frequency), with the safest profile being that of glimepiride.

Hypoglycemia is the major side effect of all sulfonylureas, while minor side effects such as headache, dizziness, nausea, hypersensitivity reactions, and weight gain are also common. Sulfonylureas are contraindicated in patients with hepatic and renal diseases and are also contraindicated in pregnant patients due to the possible prolonged hypoglycemic effect to infants. Drugs that can prolong the effect of sulfonylureas such as aspirin, allopurinol, sulfonamides, and fibrates must be used with caution to avoid hypoglycemia. Moreover, other oral antidiabetic medications or insulin can be used in combination with sulfonylurea and can substantially increase the risk of hypoglycemia.

Patients on beta-adrenergic antagonists for the management of hypertension can have hypoglycemia unawareness. Sulfonylureas should be used with caution in subjects receiving beta blockers.

#### Meglitinide

Meglitinides (repaglinide and nateglinide) are non-sulfonylurea secretagogues, which was approved as treatment for T2DM in 1997. Meglitinide shares the same mechanism as that of sulfonylureas; it also binds to the sulfonylurea receptor in β-cells of the pancreas. However, the binding of meglitinide to the receptor is weaker than sulfonylurea, and thus considered short-acting insulin secretagogues, which gives flexibility in its administration. Also, a higher blood sugar level is needed before it can stimulate β-cells’ insulin secretion, making it less effective than sulfonylurea. Rapid-acting secretagogues (meglitinides) may be used in lieu of sulfonylureas in patients with irregular meal schedules or those who develop late postprandial hypoglycemia while using a sulfonylurea.

#### Thiazolidinedione

Like biguanides, TZDs improve insulin action. Rosiglitazone and pioglitazone are representative agents. TZDs are agonists of PPAR and facilitate increased glucose uptake in numerous tissues including adipose, muscle, and liver. Mechanisms of action include diminution of free fatty acid accumulation, reduction in inflammatory cytokines, rising adiponectin levels, and preservation of β-cell integrity and function, all leading to improvement of insulin resistance and β-cell exhaustion. However, there are high concerns of risks overcoming the benefits. Namely, combined insulin-TZD therapy causes heart failure. Thus, TZDs are not preferred as first-line or even step-up therapy.

#### Other Glucose-Lowering Pharmacologic Agents

Pramlintide, an amylin analog, is an agent that delays gastric emptying, blunts pancreatic secretion of glucagon, and enhances satiety. It is a Food and Drug Administration (FDA)-approved therapy for use in adults with T1DM. Pramlintide induces weight loss and lowers insulin dose. Concurrent reduction of prandial insulin dosing is required to reduce the risk of severe hypoglycemia. Other medications that may lower blood sugar include bromocriptine, alpha-glucosidase inhibitors like voglibose and acarbose, and bile acid sequestrants like colesevelam. It may be noted that metformin sequesters bile acids in intestinal lumen and thus has a lipid-lowering effect, also the same mechanism may contribute to gas production and gastrointestinal disturbances.

#### Pharmacologic Management of Diabetes Complications

Important components of the Standards of Medical Care in Diabetes involves taking care of complications of diabetes and comorbidities including hypertension, atherosclerotic cardiovascular disease (ASCVD), dyslipidemia, hypercoagulopathy, endothelial cell dysfunction, nephropathy, and retinopathy. CVD is the most important cause of morbidity and mortality in patients with diabetes. The currently recommended goal blood pressure is ≤140/80 for patients with diabetes and hypertension. Angiotensin-converting enzyme inhibitors or angiotensin receptor blockers are the preferred antihypertensive medication (2). Optimal blood pressure and blood glucose control can effectively delay the progression of nephropathy and retinopathy in these patients. Patients with existing CVD should be continuously managed with aspirin, including providing primary prevention in subjects less than 50 years old. Patients with diabetes are also recommended to undergo annual lipid profile measurement, and those diagnosed with hyperlipidemia should be treated with statins with a low-density lipoprotein goal of <70 mg/dL (2). Moreover, it should be noted that an important aspect in the success of pharmacotherapy is patient’s adherence and compliance to medications; therefore, close and regular patient follow-up, monitoring, and education are necessary.

### Glucose Monitoring

Self-monitoring of blood glucose and HbA1C are integral components of the standards of care in diabetes. They are designed to assess the effectiveness of a treatment plan and provide guidance in selecting appropriate medications and dosage/s (2). SMBG allows patients to assess their own response to medication, minimize the risk of hypoglycemia, and determine whether they are achieving glycemic control. Optimal glycemic control is achieved when FPG is 70–130 mg/dL, 2 h post prandial <180 mg/dL, and bedtime glucose is 90–150 mg/dL. However, testing six to eight times daily may burden patients and may result in non-compliance. Therefore, it is recommended to ensure that patients are properly instructed and are given regular evaluation and follow-up.

Self-monitoring of blood glucose is essential in patients with diabetes who are on intense insulin regimen (three to four injections of basal and prandial or insulin pump). It monitors and prevents hyperglycemia and possible side effect of hypoglycemia. Blood glucose level is usually checked prior to meals, prior to exercise, prior to driving, and at bedtime. Evidence is insufficient to prescribe SMBG for patients not receiving an intensive insulin regimen (26).

According to the current guideline, HbA1C level should be assessed regularly in all patients with diabetes. The frequency of HbA1C testing is flexible and depends primarily on the response of patients to therapy and the physician’s judgment. HbA1C testing is performed at least every 6 months for patients who are meeting treatment goals; for patients who are far from their glycemic goals, HbA1C testing may be performed more frequently.

## Summary/Conclusion

Type 2 diabetes mellitus is one of the leading causes of renal failure, ASCVD, non-traumatic lower limb amputation, blindness, and death worldwide. It is a serious chronic medical condition that requires a multidisciplinary team approach, consisting of healthcare professionals, dietitians, patient educators, patients, and their families. Lifestyle intervention designed to manage body weight and treat obesity, as well as patient education, are essential for all patients with diabetes. Treatment options may be individualized and medication(s) chosen based on a patient’s risk factors, current HbA1C level, medication efficacy, ease of use, patient’s financial situation/insurance/costs, and risk of side effects such as hypoglycemia and weight gain. Effectiveness of therapy must be evaluated as frequent as possible using diagnostic blood tests (HbA1C), as well as monitoring for development of diabetic complications (e.g., retinopathy, nephropathy, neuropathy). Furthermore, aggressive efforts from physicians and motivating patients for compliance are the two important aspects of the prevention and management of diabetes. Sociocultural issues should be carefully considered. For example, during religious fasting (e.g., during the holy month of Ramadan), the use of pharmacologic agents that induce hypoglycemia should be used with care and insulin doses (for example, premix formulations) should be appropriately titrated and the patient should be educated for blood glucose monitoring and breaking of fast as needed (43).

By the year 2030, >70% of people with T2DM shall reside in developing countries (44). Primary prevention of T2DM should be an urgent public health policy. The disease predominantly affects working-age people and therefore has a counterproductive economic impact, compounded by the frequent occurrence and interaction of T2DM with infectious diseases (such as AIDS and tuberculosis) (45). Evidence from landmark T2DM prevention trials indicates that lifestyle modification is more effective, cheaper, and safer than medication and provides sustained benefits. Lifestyle modification may be promising approach to T2DM prevention in developing countries. This will be useful for many ethnic groups in the U.S. as well, such as South Asian, Latino, Pima Indians, and African-American populations, which may face socioeconomic challenges similar to what is seen in developing countries. Cost-contained strategies to identify at-risk individuals, followed by the implementation of group-based, inexpensive lifestyle interventions (“comfortably uncomfortable” life, as lived by people in blue zones), seem to be the best options for resource-constrained settings. T2DM pathophysiology is increasingly understood as a mix of insulin resistance and secretory defects of β-cells (46).

Several options for pharmacologic therapy of lowering blood glucose are currently available, which have revolutionized long-term management of DM (47). Several antidiabetic drugs may have important CV complications, which the provider team should always be aware (48). The polypharmacy issues, management of diabetes, as well as hypertension, hyperlipidemia, and use of aspirin should be carefully explained to patients to ensure adherence to therapy to prevent significant CV morbidity and mortality. Careful attention should be paid to development of insulinopenic states by clinical assessment of C peptide and lack of control of HbA1C with multiple medications, and complete lack of secreted insulin conditions should be treated by initiation of appropriate insulin regimens. Every clinical encounter should also be utilized to explain the benefit of weight loss and motivated for such. Even though not yet conclusive, clinical trial and data support consideration of bariatric surgery as a possible strategy to monitor blood glucose levels and body weight, especially in morbid obesity (49). Balanced hypocaloric diets that cause weight loss must be adopted, and regular interactions with dietitian is a useful approach. Aerobic training and resistance training can control increasing lean mass in middle-aged and overweight/obese individuals. Behavioral strategies for weight loss should be encouraged in primary care settings and appropriate maintenance of body weight prior to conception may help after development of gestational diabetes. Weight loss may be particularly challenging for incapacitated patients and subjects with disabilities, so comprehensive approaches should be undertaken. Newer molecular studies have demonstrated the transcriptional link between inflammatory pathways and increased adipose tissue storage, contributing to insulin resistance (50). Drug repurposing of the anti-inflammatory agent for aphthous stomatitis, amlexanox, is currently undergoing trials as newer agents for management of diabetes (51).

## Author Contributions

AC conceptualized and led project and drafted manuscript. CD checked accuracy of clinical contents and provided numerous clinical pearls. VSRD checked accuracy of clinical contents and numerous clinical discussions. SK contributed to numerous clinical pearls and revisions. AC contributed to important clinical discussions and revisions. RR contributed to important clinical discussions and revisions. AM contributed to important clinical contribution, especially management with coexistent chronic diseases. NSS contributed to clinical concepts and numerous clinical discussions. MTM prepared initial outline of some aspects of the manuscript. KK contributed to numerous clinical discussions. AS contributed to clinical discussions. AB checked grammar and formatted the initial table. NP contributed to initial discussions. CKM checked accuracy of clinical contents. GPL contributed to important clinical contents and numerous clinical guidance. WM contributed to overall senior mentorship and guidance and support to project.

## Conflict of Interest Statement

The authors declare that the research was conducted in the absence of any commercial or financial relationships that could be construed as a potential conflict of interest.
